# Insights and advances in chronic urticaria: a Canadian perspective

**DOI:** 10.1186/s13223-015-0072-2

**Published:** 2015-02-11

**Authors:** Gordon Sussman, Jacques Hébert, Wayne Gulliver, Charles Lynde, Susan Waserman, Amin Kanani, Moshe Ben-Shoshan, Spencer Horemans, Carly Barron, Stephen Betschel, William H Yang, Jan Dutz, Neil Shear, Gina Lacuesta, Peter Vadas, Kenneth Kobayashi, Hermenio Lima, F Estelle R Simons

**Affiliations:** University of Toronto, Medicine, Toronto, ON Canada; Université Laval, Medicine, Quebec, QC Canada; Memorial University, Medicine, Quebec, QC Canada; McMaster University Medicine, Hamilton, ON Canada; University of British Columbia, Medicine, Kelowna, BC Canada; McGill University, Medicine, Montreal, QC Canada; University of Ottawa, Medicine, Ottawa, ON Canada; Dalhousie University, Medicine, Halifax, NS Canada; University of Manitoba, Medicine, Winnipeg, MB Canada

**Keywords:** Chronic urticaria, Diagnosis, Classification, Management, Immunology, Antihistamines, Up-dosing, Omalizumab

## Abstract

In the past few years there have been significant advances which have changed the face of chronic urticaria. In this review, we aim to update physicians about clinically relevant advances in the classification, diagnosis and management of chronic urticaria that have occurred in recent years. These include clarification of the terminology used to describe and classify urticaria. We also detail the development and validation of instruments to assess urticaria and understand the impairment on quality-of-life and the morbidity caused by this disease. Additionally, the approach to management of chronic urticaria now focuses on evidence-based use of non-impairing, non-sedating H1-antihistamines given initially in standard doses and if this is not effective, in up to 4-fold doses. For urticaria refractory to H1-antihistamines, omalizumab treatment has emerged as an effective, safe option.

## Introduction

Urticaria is characterized by wheals of various sizes surrounded by flares (erythema). Mast cells play a central role in the pathophysiology. Their activation through immunologic or non-immunologic processes induces degranulation that results in release of potent preformed inflammatory mediators such as histamine, and newly-synthesized mediators such as leukotrienes and prostaglandins. The main biologic effects of these mediators are pruritus, increased vascular permeability, and tissue edema. Pruritus, the primary symptom, results from mediator-induced sensory nerve impulses travelling to the cerebrum and is associated with significant morbidity [1-3].

In this review, we provide an update on clinically relevant advances in the classification, investigations, and management of chronic urticaria that have occurred in the past few years (see list of key points for insights and advances).

### List of key points for insights and advances

- The term chronic spontaneous urticaria (CSU) replaces the term chronic idiopathic urticaria (CIU).
- Second-generation non-impairing non-sedating H1-antihistamines are recommended for first-line treatment of CSU.
- Up-dosing with second-generation non-impairing non-sedating H1-antihistamines (to 2, 3, or 4 times the licensed dose) is recommended as second-line treatment in CSU.
- Omalizumab (anti-IgE antibody) effectively and safely induces remission in H1-antihistamine-resistant CSU and is used as third-line treatment for this indication.

## Review

### Definitions, classification, and epidemiology

**Figure 1:**
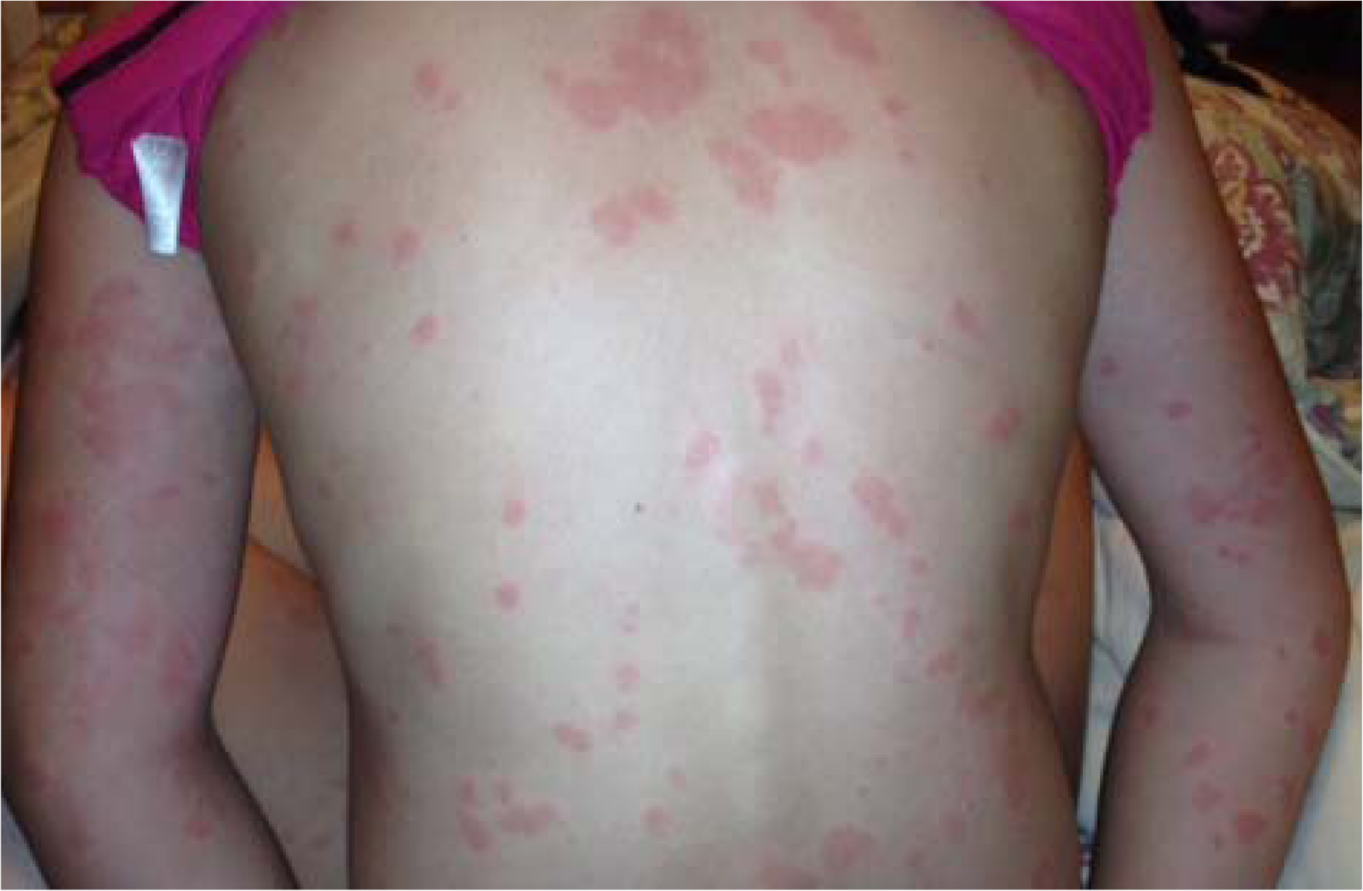
**Chronic spontaneous urticaria.**

**Figure 2:**
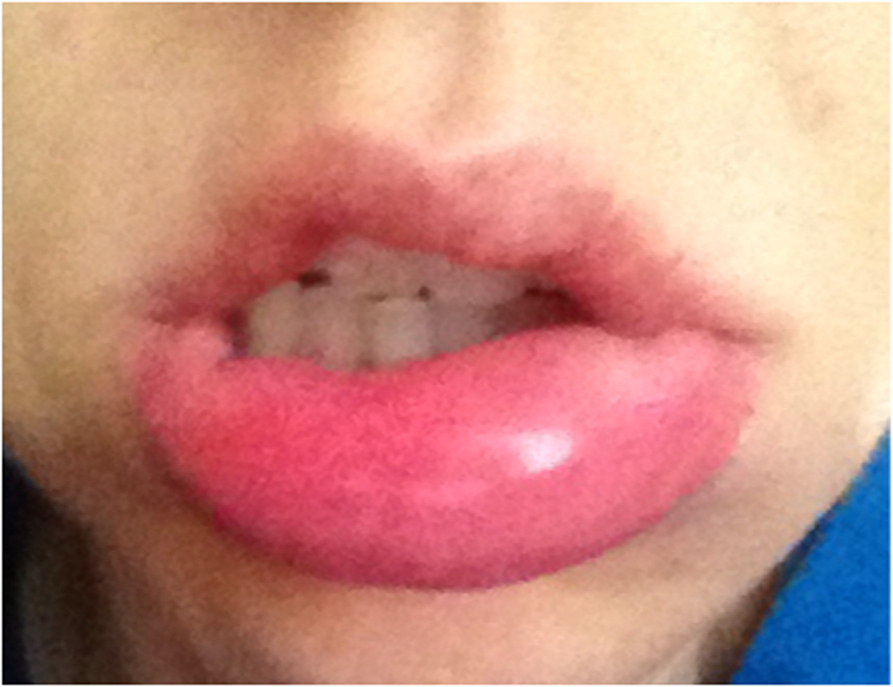
**Angioedema.**

Acute urticaria, defined as hives lasting less than 6 weeks, can occur spontaneously, or be associated with acute viral infections or allergic reactions to foods, medications, or insect stings or bites. Chronic urticaria, defined as hives occurring intermittently for at least 6 weeks, is further classified as spontaneous, i.e. chronic spontaneous urticaria (CSU), or inducible by physical stimuli (Figure 1, Table 1). Spontaneous lesions are not inducible. The prevalence of CSU is 0.5% to 1% in the general population. It occurs most commonly in women, and has a peak age of onset between 20 and 40 years. There is associated angioedema, defined as asymmetric non-dependent swelling of sub-mucosal tissues, in 33%-66% of patients with CSU [1-3] (Figure 2).

**Table 1:** **Classification of chronic urticaria subtypes (presenting with wheals, angioedema, or both)**

	**Acute urticaria**	**Chronic urticaria**
**Characteristics**	Persists for less than 6 weeks	Chronic spontaneous urticaria
		Persists for at least 6 weeks
		Inducible urticaria
		Occurs in response to an identifiable (physical) trigger.
		Subtypes include:
		● Physical urticaria (dermographism, cold urticaria, delayed pressure urticaria, solar urticaria, heat urticaria, vibratory angioedema)
		● Cholinergic urticaria
		● Contact urticaria
		● Aquagenic urticaria

### Etiology and pathogenesis

Spontaneous implies an endogenous cause; therefore, it is not surprising that in most patients with CSU, an external trigger such as an allergen cannot be identified [1,2]. Approximately 45% of patients have an autoimmune component associated with antibodies to the IgE receptor alpha subunit or to IgE itself on mast cell surfaces. Also, approximately 25% of CSU patients have associated thyroid auto-antibodies; however, in CSU, these antibodies do not correlate with altered thyroid function [1-3].

Patients with CSU can have high levels of anxiety. Psychiatric co-morbidities including mood, anxiety, and personality disorders have been reported [4]. These co-morbidities are often major contributors to quality of life impairment [5]. It has been suggested that psychosocial factors may play a role in the pathogenesis of CSU [4,6].

### Disease course and prognosis

CSU, by definition, persists for at least six weeks and its course is typically characterized by exacerbations and remissions. From 10% to 50% of patients have CSU for longer than 5 years. Longer duration of illness is associated with severe disease. Elevated plasma levels of C-reactive protein, prothrombin 1 and 2, and D-dimer are reported to function as markers of CSU severity [7].

### Practice guidelines

Urticaria guidelines published in 2014 include new information on CSU classification, investigations, instruments available to grade severity and impact on quality of life, as well as new recommendations for management [2,3].

### Investigations

Diagnosis of CSU is based on a thorough history and physical examination. Specific questioning about use of non-steroidal anti-inflammatory drugs(NSAIDs)is important because up to 30% to 50% of CSU patients have exacerbations associated with NSAID ingestion [1,2]. All patients require a complete physical examination, including visualization and confirmation of characteristic pruritic, raised erythematous lesions (Figure 1). Serial photographs are useful for documenting the extent and severity of the urticaria. The frequency, pattern, and duration of lesions should be recorded.

**Figure 3:**
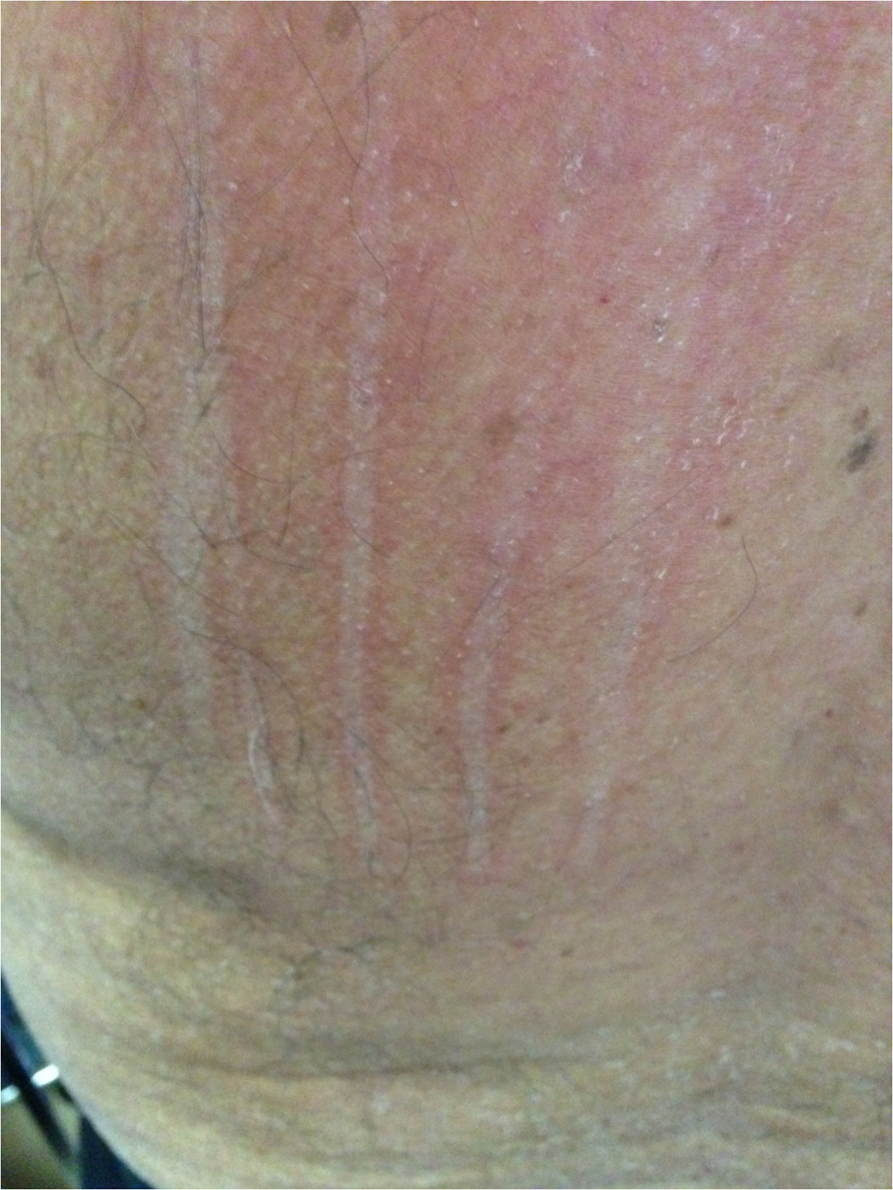
**Dermographism.**

**Figure 4:**
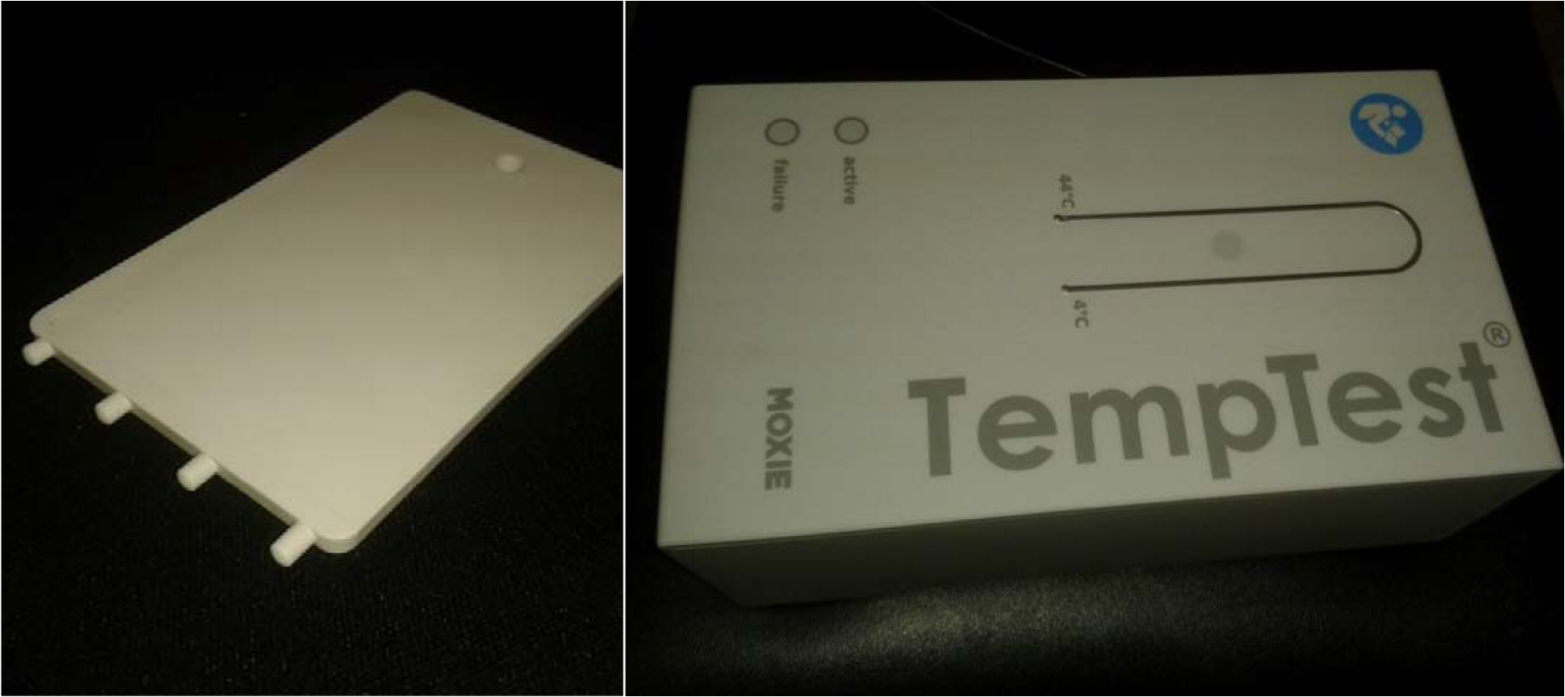
**The dermographometer, or FricTest®, (left), a standardized instrument for diagnosis of dermatographic urticaria and the TempTest® (right) a new tool for diagnosing cold urticaria.** Both instruments are produced by Moxie GmbH (Berlin, Germany).

Inducible urticarial lesions typically persist for less than two hours, with the exception of delayed pressure urticaria, which can last for more than 24 hours. Patients with CSU should be tested for dermographic urticaria using a tongue depressor or, if available, a standardized instrument, to stroke the skin firmly and induce wheal formation (Figure 3). Physical challenge tests for cold-induced urticaria using an ice cube or, if available, a standardized instrument, or tests for other subtypes of inducible urticaria, should be considered in patients with a history of exacerbations from these stimuli (Figure 4, Table 2).

**Table 2:** **Recommended investigations in urticaria according to type and subtype**

**Type**	**Subtype**	**Initial tests**	**More extensive tests**
Spontaneous urticaria	Acute spontaneous urticaria	None	None*
	Chronic spontaneous urticaria	CBC with differential and ESR or CRP	(i) Allergen skin testing, and measurement of allergen-specific IgE levels are seldom required in CSU. Measurement of IgG levels to foods has no diagnostic value. (ii) functional autoantibodies; (iii) thyroid hormones and autoantibodies; (iv) physical tests; (v) tryptase; (vi) autologous serum skin test; (vii) lesional skin biopsy
Inducible urticaria	Cold urticaria	Cold provocation and threshold test: apply an ice cube to the skin for 5 min, or, if available, use a TempTest; urticaria appears on re-warming	CBC with differential and ESR/CRP cryoproteins
	Delayed pressure urticaria	Pressure test	None
	Heat urticaria	Heat provocation and threshold test	None
	Solar urticaria	UV and visible light of different wave lengths	Rule out other light-induced dermatoses
	Symptomatic dermographism	Elicit dermographism by stroking skin firmly with a tongue depressor or, if available, use a FricTest	None
	Aquagenic urticaria	Wet cloths at body temperature applied for 20 min	None
	Cholinergic urticaria	Exercise and hot bath provocation	None
	Contact urticaria		None

CBC, complete blood count; ESR, erythrocyte sedimentation rate; CRP, C-reactive protein; NSAID, non-steroidal anti-inflammatory drug.

*Acute urticaria and angioedema can also occur in the context of anaphylaxis. Such patients should be tested to allergens relevant to the history of their anaphylactic episode, eg. foods, stinging insect venoms or medications (references [2,3]).

Guidelines now recommend that the initial investigation of CSU should generally be limited to a complete blood count (CBC) and differential, and measurement of inflammation markers such as erythrocyte sedimentation rate (ESR) or C-reactive protein (CRP) [2,3] (Table 2).

Depending on the history, other investigations may be indicated (Table 2). A skin biopsy should be performed in patients with atypical urticaria; for example, those with burning or painful hives that persist for longer than 72 hours, suggestive of urticarial vasculitis. Patients with hyperpigmented lesions should have the skin stroked firmly to elicit Darier’s sign (whealing) suggestive of cutaneous mastocytosis and a baseline serum tryptase level to rule out mastocytosis; a lesional biopsy should be performed if indicated. Allergy skin tests generally have limited diagnostic value. The autologous serum skin test (ASST), performed by intradermal injection of autologous serum using careful sterile technique, is rarely used in practice. A positive ASST suggests the presence of auto-antibodies to the high-affinity IgE receptor or to IgE; however, this test is not specific for CSU [7]. Assays for auto-antibodies to the high-affinity IgE receptor or to IgE are only available in specialized research laboratories. Newer tests for auto-antibodies are being developed.

### Impact on quality of life

CSU has been shown to cause significant morbidity and to have a negative impact on all aspects of a patient’s life, including work, school, social activities, diet and sleep. In an attempt to quantify this, new instruments have been developed to grade urticaria severity. The Urticaria Activity Score, recorded daily for a week (UAS7), is comprised of an extent of wheals score and a pruritus score (Table 3). A UAS7 score of 6 or less is generally considered to indicate well controlled chronic urticaria. The impact of urticaria on quality of life can be assessed by a Quality of Life questionnaire (CU-Q2oL) [8], which has been validated in Canada in both English and French. Validated angioedema activity scores and a QoL score for angioedema are also available and are important tools for assessing the disability associated with disfiguring angioedema [9]. Recently, the urticaria control test (UCT) has been validated for grading severity of urticaria. This tool permits the assessment of chronic urticaria, angioedema, and inducible urticaria [10].

**Table 3:** **The Urticaria Activity Score (UAS7) for assessing disease activity in chronic spontaneous urticaria**

**Score**	**Wheals**	**Pruritus**
0	None	None
1	Mild (<20 wheals/24 h)	Mild (present but not annoying or troublesome)
2	Moderate (20–50 wheals/24 h)	Moderate (troublesome but does not interfere with normal daily activity or sleep)
3	Intense (>50 wheals/24 h or large confluent areas of wheals)	Intense (severe pruritus, which is sufficiently troublesome to interfere with normal daily activity or sleep)

### Management

The overall goal is complete symptom control and disease remission. Factors that exacerbate CSU, including stress and ingestion of NSAIDs should be avoided. Second-generation non-impairing non-sedating H_1_-antihistamines, which are effective, safe, and inexpensive, are recommended as first-line treatment. They should be taken on a regular daily basis, not on an as-needed basis. There is no advantage in using more than one H_1_-antihistamine at the same time (Table 4) [2,3,11,12].

**Table 4:** **Urticaria treatment algorithm**

**First line**	
Second-generation non-impairing non-sedating antihistamines *if symptoms persist after 2 weeks*	Standard dosing. Desloratadine 5 mg OD. Loratadine 10 mg OD. Cetirizine HCI 10 mg OD. Fexofenadine HCI 60 mg BID
↓	
**Second-line**	
Increase dosage up to four-times the standard dose^1,2^ of a second-generation non-impairing non-sedating antihistamine *or, if symptoms persist after 4 further weeks,* add montelukast for a 3–4 week trial	Up-dosing to the limit specified, eg. Desloratadine up to 20 mg OD. Cetirizine HCI up to 40 mg OD^3^. Montelukast 10 mg OD
Exacerbation: oral corticosteroid	**Re-evaluate response to treatment every 3 months**
↓	Omalizumab 150 mg or 300 mg, SC Q4 wks. Cyclosporine A 2.5-5 mg/kg/day and taper with response
**Third line**	Oral corticosteroids, for example, 0.3-0.5 mg/kg of prednisone or equivalent, followed by tapering of the dose in half every 3–7 days over a maximum duration of 2–4 weeks
Add-on to second-line treatment: omalizumab, cyclosporine A, consider specialist referral to allergist/dermatologist. Exacerbation: oral corticosteroid	

^1^Standard dose means the usual recommended dose.

^2^Double the initial recommended pediatric dose in case of non-response.

^3^Can cause sedation at these doses.

Up-dosing with two, three, or four times the licensed dosage, for example, desloratadine up to 20 mg, is suggested as second-line treatment to be implemented if the licensed dosage of an H_1_-antihistamine is not effective [13-15].

First-generation sedating impairing H_1_-antihistamines are no longer recommended for use in urticaria, because they potentially interfere with restful (rapid eye movement) sleep, cause hangovers or “morning-after” effects, impair learning and memory, and reduce work efficiency. Impairment is not necessarily accompanied by sedation [10,16].

Montelukast can be used as add-on second-line treatment in H_1_-antihistamine refractory CSU, although some clinical trials have not shown a significant benefit [17]. H_2_-antihistamines are no longer recommended as first-, second-, or third-line treatment, as the level of evidence supporting their use is low [18].

At any stage of treatment, a short course of an oral corticosteroid can be added on an empirical basis to provide rescue treatment of a CSU exacerbation. One example of a suggested corticosteroid treatment regimen is an initial dose of 0.3 – 0.5 mg/kg of prednisone or equivalent, followed by tapering of the dose in half every 3–7 days over a maximum duration of 2–4 weeks [19].

Recommended third-line treatments include omalizumab, or off-label use of cyclosporine A [2,3,17,20-25].

A trial of cyclosporine A is recommended as add-on treatment to a second-generation H_1_-antihistamine in patients who fail to respond adequately to treatment with an H_1_-antihistamine alone (high level of evidence). Administered in a dose of 3–5 mg/kg divided twice daily, cyclosporine can lead to complete remission of urticaria; however, regular monitoring of blood pressure, complete blood count, and renal function, followed by dose adjustment when needed, is advised in order to reduce the possibility of toxicity [17].

Omalizumab, an anti-IgE humanized monoclonal antibody, can be used as add-on therapy to a non-sedating second-generation H_1_-antihistamine as a third-line treatment for CSU (high level of evidence) [20,21]. Its efficacy and safety have been demonstrated in large, well-designed, randomized double-blind, placebo-controlled trials in patients with CSU refractory to H_1_-antihistamine treatment [20,21]. Some studies suggest that 300 mg omalizumab injected subcutaneously every four weeks is the effective dose; however, others suggest that 150 mg is effective [21]. Additional investigations are needed to define the optimal dose, dose schedule, and duration of treatment [24].These issues are important from a cost-effectiveness perspective. In 2014, in Canada, the United States and in Europe, omalizumab was approved by regulatory agencies for treatment of H_1_-antihistamine-refractory chronic urticaria in adults and adolescents age 12 years and older [25].

Use of other pharmacologic agents as third-line treatments, including hydroxychloroquine, dapsone, mycophenolate mofentil, tacrolimus, intravenous gamma-globulin and sulfasalazine, is based mainly on case reports and uncontrolled trials [15].

CSU is self-limited and eventually remits completely in most patients. Until remission occurs, whether this takes months, years, or decades, the therapeutic goal remains complete relief of itching and other symptoms.

## Conclusions

The urticarias are a heterogeneous group of disorders, classified as acute or chronic (which can be spontaneous or inducible). In the new terminology, the term “spontaneous” replaces the term “idiopathic” and the term “inducible” replaces the term “physical”. Chronic spontaneous urticaria (CSU) affects 0.5% to 1% of the population, and has a negative impact on all aspects of quality of life.

After the correct clinical diagnosis is made, most patients need only limited testing. Careful history, physical examination, and follow-up will identify those who require additional investigations.

Second-generation non-impairing non-sedating H_1_-antihistamines are recommended as first-line medications for initial treatment. New effective and safe therapeutic options have emerged for treatment of patients with CSU refractory to the standard dosage of an H_1_-antihistamine. Up-dosing with a second-generation non-impairing non-sedating H_1_-antihistamine such as desloratadine in 2, 3, or 4 times the licensed dosage is recommended as second-line treatment. Omalizumab injections subcutaneously at monthly intervals are recommended as a novel effective and safe therapeutic option for CSU refractory to the above. Research in progress will help to define some specific aspects of these new approaches and further establish their place in the treatment algorithm.

## Consent

Written informed consent was obtained for publication of the accompanying images in this review. A copy of the written consent is available for review by the Editor-in-Chief of this journal.

## Abbreviations

CSU: Chronic spontaneous urticaria
NSAIDS: Non-steroidal anti-inflammatory drugs
CBC: Complete blood count
ESR: Erythrocyte sedimentation rate
CRP: C-reactive protein
ASST: Autologous serum skin test
UAS7: Urticaria Activity score recorded daily for a week
CU-Q2oL: Quality of Life questionnaire
UTC: Urticaria control test
